# Suspected Enhanced S-Cone Syndrome: A Case Report

**DOI:** 10.7759/cureus.43660

**Published:** 2023-08-17

**Authors:** Ghadah Alnosair, Rabab Aljayani

**Affiliations:** 1 Pediatric Ophthalmology, Dammam Medical Complex, Dammam, SAU; 2 General Medicine, Dammam Medical Complex, Dammam, SAU

**Keywords:** retinal dystrophy, enhanced s-cone syndrome, diagnosis, electroretinography, case report

## Abstract

Enhanced S-cone syndrome (ESCS) is a rare type of retinal dystrophy disorder that is linked to NR2E3 gene mutation and NRL gene mutations less widely. The disease is characterized by increased S-cones number and marked degeneration in rods and M- and L-cone receptors. The patient suffers from night blindness from an early age. Examination of the fundus of the eye shows nummular pigmented lesions, but they are not specific to ESCS. The diagnosis can be confirmed with electroretinography. We report a case of a four-year-old girl suspected of having ESCS based on her clinical picture, fundus examination, and electroretinography.

## Introduction

Enhanced S-cone syndrome (ESCS) is a rare, autosomal recessive, slowly progressive retinal dystrophy. In this disorder, the short-wavelength-sensitive cone photoreceptors are hypersensitive. On the other hand, rods, M-, and L-cone receptors are degenerated [1].

Patients with ESCS are highly sensitive to blue light. Nyctalopia from an early age is the main presenting symptom. Mutation in NR2E3 gene is implicated in the pathogenesis. This gene differentially regulates the transcription of rod-specific and cone-specific genes. This mutation leads to overexpression of S-cones but suppression of L-cone and M-cone expression [2].

ESCS may be misdiagnosed as other retinal dystrophies, such as retinitis pigmentosa and congenital stationary night blindness. Fundus examination in ESCS shows characteristic nummular pigmentary changes at the level of the retinal pigment epithelium along the vascular arcades, and sometimes in the posterior pole, and these can then progress to the more classic pigmented lesions [1]. Other reported findings include cystoid maculopathy, foveal, and peripheral schisis. Uncommon findings include subretinal fibrosis and choroidal neovascularization, in addition, oval torpedo-like lesions with a central area of pigmented epithelium depigmentation and a hyperpigmented margin [3,4].

Differentiation from other retinal dystrophies can be made with a pathognomonic response on electroretinogram (ERG). Characteristic ERG findings include the absence of rod response on low-intensity, dark-adapted stimulus, and a simplified, delayed waveform at high-intensity, dark-adapted stimulus besides a similar waveform in both scotopic and photopic conditions. The 30-Hz flicker responses are markedly delayed with lower amplitude [5].

Here, we present a case of a four-year-old girl suspected of having ESCS. Her mother reported that the girl had diminished night vision. Her fundus examination and ERG findings strongly supported the diagnosis.

## Case presentation

**Figure 1:**
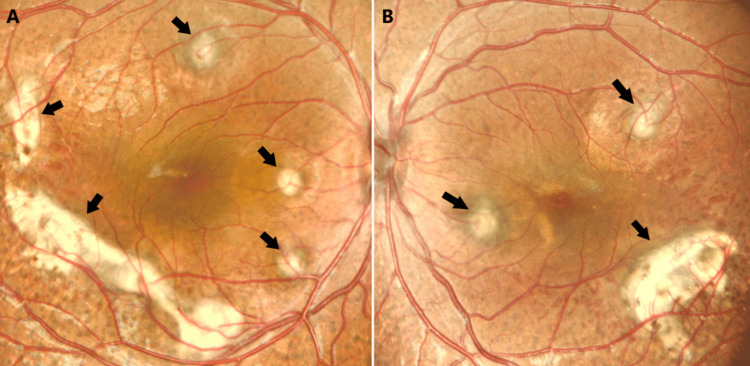
Colored fundus photos of the right (a) and left (b) eyes showing bilateral multiple deep yellowish choroidal lesions (arrows). The optic nerve head and vessels are within normal limits.

A four-year-old girl had been suffering from visual difficulties for four months. Her mother noticed that the girl had poor visual acuity at night. Her parents were consanguineous. The child had no relevant systemic diseases. Initially, visual acuity of 0.4 was recorded in both eyes with glasses. Cycloplegic refraction of +7.00 and +7.25 with glasses was recorded in the right and left eyes, respectively. Clinical features were bilateral and mostly symmetrical. Slit-lamp examination showed that the corneas and the anterior chambers were normal with clear lenses. Fundus examination revealed bilateral, multiple, deep yellowish, choroidal lesions with a punched-out appearance. Some of these lesions were pigmented. The lesions involving the macula were large and fibrosed. The vitreous was clear as shown in color fundus photos (Figure 1).

**Figure 2:**
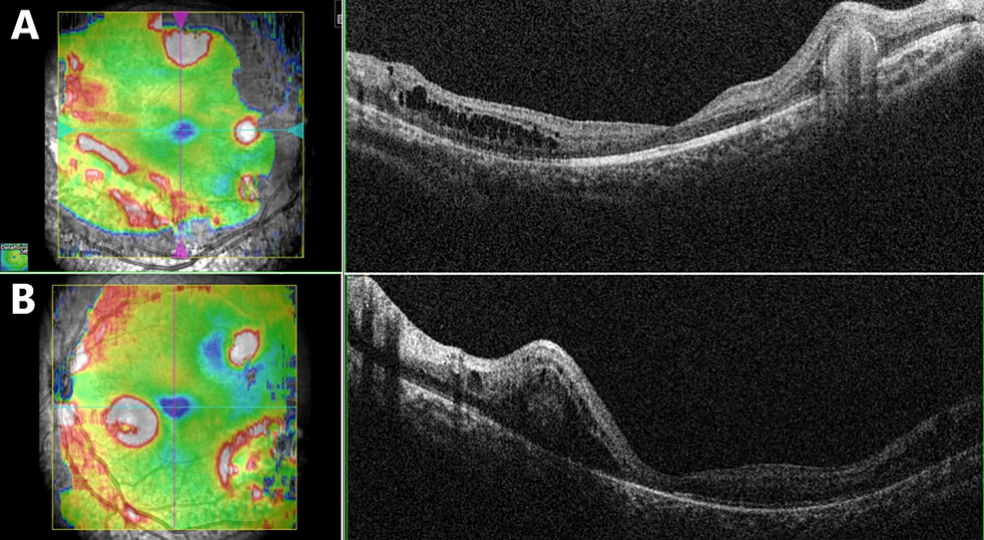
Optical coherence tomography of the right (a) and left (b) eyes showing multiple, round, subretinal, hyperreflective accumulations. Some of these accumulations were associated with intraretinal fluids and cysts with no foveoschisis.

Blood investigations for toxoplasmosis immunoglobulin-G and immunoglobulin-M were negative. Testing for antinuclear antibodies was positive (+2). Diminished rod and cone response and diminished flicker response were found on ERG. Optical coherence tomography of the macula showed multiple, round, subretinal, hyperreflective accumulations. Some of these accumulations were associated with intraretinal fluids and cysts (Figure 2).

Fundus autofluorescence reveals decreased autofluorescence in the peripheral retina with increased autofluorescence in the macula. The yellow-white lesions are hyperautofluorescent.

## Discussion

We report a case of a four-year-old girl suspected of having ESCS. The mother noticed that the child had been suffering from nyctalopia for four months prior to the presentation. Although ESCS is a rare retinal disorder, the presence of yellowish, choroidal lesions on fundus examination raised the suspicion of ESCS. There is a wide range of fundus findings of ESCS. However, the typical appearance is nummular pigmented deposits at the level of retinal pigment epithelium and intraretinal cysts. Yellow pigmentation in the mid-peripheral fundus was reported by Naik et al. as the most common fundus finding [1].

Yzer et al. noted that all patients who had subretinal fibrosis had yellow retinal dots as well [4]. In addition to the fundus findings, the age of the child and nyctalopia as the main presenting symptom are consistent with the literature. The usual age of presentation has been reported to be the first two decades of life [2]. Despite Naik et al. reported that only five patients in their series presented in their first decade, previous misdiagnoses of other patients as having other types of retinal dystrophies probably caused the delay in presentation [1].

A large range of visual affection has been reported in ESCS cases, ranging from normal to severely diminished visual acuity [2,4]. In our patients, the macular lesions were fibrosed. Alsalamah et al. conducted a recent retrospective study on 101 patients with ESCS [6]. The researchers found macular subretinal fibrosis in 47 of the enrolled patients. Cystic changes in the macula were reported as common findings in earlier studies, which is consistent with our findings [7,8].

Nummular pigmentary deposition alone is not specific to ESCS, and it has been described in other retinal dystrophies [2,9]. The pathognomonic features of ERG in ESCS are well established. Our patient's ERG showed normal rod response with delayed, diminished flicker response. The full-field ERG amplitude in molecularly confirmed ESCS cases varies from being abnormally large to severely reduced. There were reports of preserved rod function [10]. de Carvalho et al. in their cohort study considered the presence of clinical features suggestive of ESCS alongside typical electroretinography enough to diagnose ESCS irrespective of molecular confirmation [2].

## Conclusions

The rarity of ESCS can pose a challenge in its diagnosis if there is a lack of awareness of the condition. There is a wide spectrum of functional and structural abnormalities of the retina in ESCS. A pathognomonic ERG is essential to make an accurate diagnosis. Documented gene mutation can be further proof.

Nyctalopia at a young age and nummular pigmented lesions in the retina should raise suspicion and impose further investigations. Recognition of this slowly progressive condition is important since it has a different prognosis from other phenotypically similar disorders.
